# The Concept of Health-Promoting Collaboration—A Starting Point to Reduce Presenteeism?

**DOI:** 10.3389/fpsyg.2021.782597

**Published:** 2022-01-13

**Authors:** Rebecca Komp, Simone Kauffeld, Patrizia Ianiro-Dahm

**Affiliations:** ^1^Department of Management Sciences, Bonn-Rhein-Sieg University of Applied Sciences, Rheinbach, Germany; ^2^Department of Industrial/Organizational and Social Psychology, Institute of Psychology, Technical University Braunschweig, Braunschweig, Germany

**Keywords:** health-promoting collaboration, presenteeism, well-being, work ability, health management

## Abstract

**Background:** Since presenteeism is related to numerous negative health and work-related effects, measures are required to reduce it. There are initial indications that how an organization deals with health has a decisive influence on employees’ presenteeism behavior.

**Aims:** The concept of health-promoting collaboration was developed on the basis of these indications. As an extension of healthy leadership it includes not only the leader but also co-workers. In modern forms of collaboration, leaders cannot be assigned sole responsibility for employees’ health, since the leader is often hardly visible (digital leadership) or there is no longer a clear leader (shared leadership). The study examines the concept of health-promoting collaboration in relation to presenteeism. Relationships between health-promoting collaboration, well-being and work ability are also in focus, regarding presenteeism as a mediator.

**Methods:** The data comprise the findings of a quantitative survey of 308 employees at a German university of applied sciences. Correlation and mediator analyses were conducted.

**Results:** The results show a significant negative relationship between health-promoting collaboration and presenteeism. Significant positive relationships were found between health-promoting collaboration and both well-being and work ability. Presenteeism was identified as a mediator of these relationships.

**Conclusion:** The relevance of health-promoting collaboration in reducing presenteeism was demonstrated and various starting points for practice were proposed. Future studies should investigate further this newly developed concept in relation to presenteeism.

## Introduction

Going to work despite being ill seems to have become a habit for many employees. In various studies, over two-thirds of employees reported having done this more than once within a year (Aronsson and Gustafsson, 2005; Hansen and Andersen, 2008; Gustafsson and Marklund, 2011). Moreover, the trend toward increased telework is likely to result in employees working more often despite illness because they can work from home (Steidelmüller et al., 2020).

The term for the phenomenon of working while sick is presenteeism. It originated in reference to the opposite term of absenteeism, which describes absence from work (Hägerbäumer, 2017). The negative consequences of presenteeism affect both the employees themselves and the organization as a whole. On the one hand, employees have poorer health overall and more physical complaints in the long term (Aronsson et al., 2000; Gustafsson and Marklund, 2011), combined with a greater likelihood of future absenteeism due to illness (Bergström et al., 2009; Gustafsson and Marklund, 2011). On the other hand, presenteeism results in impaired work ability (Dellve et al., 2011; Gustafsson and Marklund, 2011) and productivity losses, entailing high costs (Warren et al., 2011). Furthermore, co-workers may become infected (Böcken et al., 2009).

The question arises as to what measures the organization can take to reduce presenteeism. Research has shown that workplace health promotion can reduce presenteeism (Zok, 2008; Hägerbäumer, 2017). The reductions occur because the measures taken lead to an immediate improvement in overall health. Additionally, the perceived appreciation of health may result in sensitized behavior when employees are ill (Hägerbäumer, 2017). Chen et al. (2015) also showed that presenteeism varied significantly by the level of perceived workplace health support, for example support for physical activity. Moreover, the promotion of a health culture (Hägerbäumer, 2017) and support for an organizational climate conducive to health (Komp et al., 2021) are associated with lower levels of presenteeism. A recent study identified the type of attendance culture (the extent to which presenteeism and absenteeism are condoned in the organization) as a key factor influencing employees’ decisions to work when sick (Ruhle and Süß, 2020). It has also been shown that in companies where the level of organizational health literacy is high, presenteeism is significantly lower than in companies with low levels of organizational health literacy. Organizational health literacy is the ability of an organization to maintain and promote the health of its members by creating appropriate conditions and resources (Wieland and Hammes, 2010). Overall, the organization’s approach to dealing with health seems to be a relevant starting point for reducing presenteeism.

“Healthy leadership”^1^ has been shown to be crucial in this context and has been researched in recent psychological studies (Pundt and Felfe, 2017). However, this alone does not suffice; the role of all concerned with internal collaboration (co-workers, team members) merits attention. This concept of “health-promoting collaboration” has not so far been studied in this form.

Some recent developments advocate measuring health-promoting collaboration beyond healthy leadership. In modern work contexts, teleworking is often a part of everyday life. There are more virtual teams, thus exchanges between leaders and colleagues occur increasingly remotely rather than face to face (Ohlbrecht and Seltrecht, 2018). Another point is the prevalence of shared leadership. The role of the leader typically recedes while leadership roles and influence are distributed among the team (Zhu et al., 2018). Traditional teamwork and employee-leader relationships are breaking down. In these changing conditions, it is important to examine how health can be addressed. In modern forms of teamwork, the leader is no longer the only team member responsible for employees’ health.

The developments described above also occur at universities, making the investigation of health-promoting collaboration beyond healthy leadership a relevant field of research in that context. Due to increased opportunities to work at home, exchanges within a team often take place digitally rather than in person. Since the COVID-19 pandemic, this trend has proliferated, and people have been working almost exclusively from home for months (Dittler and Kreidl, 2021). Close contact between leaders and subordinates working together in an office is no longer an option for those working remotely. Furthermore, some kind of shared leadership is often present, because, for example, at the university investigated here, there are several team leaders in many departments in addition to the leader proper. Another special characteristic in academia is the heterogeneous leadership structures due to the different groups of scientific and administrative staff. For these reasons, the sweeping assumption that leaders bear sole responsibility for the health of their subordinates needs to be reconsidered. Co-workers also play a role in implementing a health-promoting culture, as this must be lived by all employees (Osterspey, 2012). Besides the relevance of focusing on health-promoting collaboration at universities, there are also reasons why universities are interesting with regard to presenteeism: Universities often lack substitution arrangements and thus clear structures when an employee is sick (Komp et al., 2021). Moreover, many employees are on temporary contracts, possibly leading to an increase in presenteeism due to fear of job loss (Böcken et al., 2009). The general structure with timed semesters, courses, and exams means that many tasks (including administrative tasks) cannot be handled flexibly but must be accomplished at specific times. This may also serve to increase presenteeism, so that, for example, a lecture need not be canceled (Komp et al., 2021).

The present study is intended to further investigate presenteeism and, above all, ways to reduce it. The aim is not only to find approaches as to what the individual can do but also to focus on working conditions. For this purpose, the concept of health-promoting collaboration is developed, which, in contrast to healthy leadership, focuses additionally on the role of co-workers. These are also considered relevant as regards accepting responsibility for employee health. The hypotheses are presented in the following section.

## Hypotheses

### Correlation Hypotheses

As described in the preceding section, presenteeism has far-reaching negative consequences. Addressing health at the organizational level has been identified as a relevant starting point to reduce presenteeism (Wieland and Hammes, 2010; Chen et al., 2015; Hägerbäumer, 2017; Ruhle and Süß, 2020; Komp et al., 2021). We assume that, in addition to the leaders, the employees themselves are relevant in achieving a healthy working culture. This relevance was the starting point for developing the concept of health-promoting collaboration as an extension of healthy leadership. Hence to establish the assumed relationship to presenteeism, the influence of leadership must initially be considered.

Leaders can influence employees’ presenteeism behavior, particularly through capacity as role models (Dietz et al., 2020; Komp et al., 2021). Leaders themselves exhibiting presenteeism are likely to exacerbate presenteeism among employees (Dietz et al., 2020), who may assume that this behavior is equally expected of them. Furthermore, leaders should send sick employees home to demonstrate that this behavior is not desired. This attitude should also be addressed in dialogue with employees (Komp et al., 2021). All these aspects can be disseminated to co-workers. First of all, in addition to the leader, co-workers themselves serve as role models and should be aware of this with regard to presenteeism. It is also assumed that presenteeism can be reduced if employees exhibiting presenteeism are reprimanded by peers. Therefore, the following hypothesis is proposed:

Hypothesis 1: There is a negative relationship between perceptions of health-promoting collaboration and exhibiting presenteeism.

Besides this relationship to the specific construct of presenteeism, attention should be paid to whether the concept of health-promoting collaboration affects the constructs of well-being and work ability, which are much studied in the work context. Once again, studies on healthy leadership form the basis for the derivation of the hypotheses. First, healthy leadership contributes to improved employee well-being (Franke et al., 2014; Rigotti et al., 2014; Santa Maria et al., 2019). Rigotti et al. (2014) investigated the impact of healthy leadership on various indicators of well-being. They detected significant effects in each of the expected directions across multiple measurement time points. It is assumed that co-workers can also have an influence on well-being. One example is the support co-workers provide when taking over tasks in stressful work phases (Osterspey, 2012). Accordingly, the following hypothesis is proposed for health-promoting collaboration:

Hypothesis 2: There is a positive relationship between perceptions of health-promoting collaboration and well-being.

Beyond well-being, healthy leadership also has an impact on employees’ work ability, which has economic implications for the organization. A positive relationship between healthy leadership and work ability has been identified (Vincent, 2011). The same mechanism of action is assumed for health-promoting collaboration because co-workers can also influence the work ability. For example, co-workers can support each other in work-related problems or generally benefit from the knowledge and experience of other team members (Osterspey, 2012) thereby improving their own work ability. The corresponding hypothesis for health-promoting collaboration is:

Hypothesis 3: There is a positive relationship between perceptions of health-promoting collaboration and work ability.

### Mediator Hypotheses

To derive practical measures, it is important to gain a comprehensive understanding of the mode of action and reciprocal influence of the two constructs, presenteeism and health-promoting collaboration.

The two constructs have in common that both influence well-being and work ability. A relationship between health-promoting collaboration and well-being and work ability is assumed (see Hypotheses 2 and 3). Presenteeism also influences both of these variables. Gustafsson and Marklund (2011) showed that individuals who went to work despite illness at least twice in the previous year had poorer psychological well-being, and Karimi et al. (2015) similarly found that presenteeism significantly predicted well-being. Dellve et al. (2011) and Gustafsson and Marklund (2011) found that presenteeism is a predictor of impaired future work ability and, consequently, of impaired performance at work.

Early studies were able to demonstrate a mediator effect of presenteeism in the work context. Dietz et al. (2020) showed that leader presenteeism has a positive indirect effect on employees’ sick leaves. This relationship is mediated by employees’ presenteeism behavior. Baeriswyl et al. (2017) were also able to show the mediating effect of presenteeism: The negative effect of co-worker support on emotional exhaustion is mediated by presenteeism. Based on these findings, the present study also assumes a mediating effect of presenteeism. In reference to the study by Baeriswyl et al. (2017), there is overlap in the constructs examined: Health-promoting collaboration also implies, but goes beyond, aspects of co-worker support. Health-promoting collaboration is hypothesized to be the predictor of our model. Emotional exhaustion as a core dimension of burnout and thus as an indicator of poor mental health is closely related to well-being. Moreover, work ability is assumed to be another criterion. Presenteeism is assumed to act as a mediator.

The idea is that the relationship between health-promoting collaboration and well-being or work ability is due, among other things, to the fact that a low level of health-promoting collaboration leads to more presenteeism, which has a negative impact on well-being or work ability. Presenteeism will be used to at least partially explain the relationships. Accordingly, the following hypotheses are proposed:

Hypothesis 2a: The positive relationship between perceptions of health-promoting collaboration and well-being is mediated by presenteeism.
Hypothesis 3a: The positive relationship between perceptions of health-promoting collaboration and work ability is mediated by presenteeism.

## Materials and Methods

### Procedure and Participants

Data collection took place in October 2019. For 3 weeks, the 734 employees of a German university of applied sciences were able to participate in a university-wide health survey. The online questionnaire was completed by 308 employees, resulting in a response rate of approximately 42%. The study was duly approved by the Ethics Committee of the Medical Faculty of the University of Bonn.

With regard to demographic data, only the following characteristics could be collected due to considerations of anonymity: Leadership position, occupational group, age group. Ten per cent of the respondents were leaders. The remaining 90% were able to further specify their occupational group. Ninety per cent were scientific or administrative employees and 10% were professors. With regard to age, a balanced picture emerged. The proportion of those under 40 was 51% and those aged 40 or older accounted for 49%. Gender could not be surveyed.

With regard to representativeness, the composition of the participants in the survey largely corresponds to the characteristics of the population at the university studied. Only older employees tended to participate somewhat less frequently in the survey (in the population 60% are 40 years or older). Consequently, it can be assumed that the participants reflect the population well. At least *N* = 303 participants responded to all items (see “Results” section). The outcome data can thus be considered to be complete.

### Measures

The scales used to survey the various constructs are described below. It should be noted that the scaling differs in each case as the original scales with the corresponding response options were used.

#### Presenteeism

Presenteeism was assessed by five items from the Gesundheitsmonitor (Bertelsmann Stiftung; Böcken et al., 2009). Although 1-item solutions are usually used, especially in European research (Lohaus and Habermann, 2019), more detailed information should be elicited here. Instead of only knowing how often presenteeism occurred, the items used here can additionally reveal the severity. The items, such as “In the last 12 months, how often did you go to work against the doctor’s advice?” could be rated on a 3-point scale (1 = “not once,” 2 = “once,” 3 = “twice or more”).

#### Well-Being

Psychological well-being was measured using the German version of the WHO-5 well-being index (Brähler et al., 2007). The scale consisted of five positively formulated items, such as “Over the past 2 weeks I have felt calm and relaxed.” The response categories ranged from 1 = “at no time” to 6 = “all the time.”

#### Work Ability

The four items used to assess work ability were taken from the DEGS questionnaire. The Study of Adult Health in Germany is conducted as part of the health monitoring of the Robert Koch Institute (Gößwald et al., 2012). For example, respondents were asked to rate the item “I could not work as long as usual” on a 5-point Likert-type scale (1 = “never” to 5 = “always”).

#### Health-Promoting Collaboration

Based on the Health-oriented Leadership Questionnaire (HoL) by Pundt and Felfe (2017), the scale to measure health-promoting collaboration was developed. The HoL differentiates between the areas StaffCare (how leaders deal with their employees with regard to health) and SelfCare (dealing with one’s own health, both in regard to the leaders and the employees). StaffCare and SelfCare each comprise three components, namely value, awareness, and behavior. Value refers to the importance of one’s own health and that of the employees. Awareness includes the conscious perception of one’s own state of health and the stress level or those of the employees. The last dimension, behavior, describes actually carrying out health-promoting activities or motivating employees to do so (Pundt and Felfe, 2017).

The items used here were slightly modified from the StaffCare section of the questionnaire for employees. The SelfCare section was not implemented because the focus was on collegial collaboration and not on dealing with one’s own health. The main difference between the HoL and the items used here is that the HoL has a clear focus (solely) on the leaders, but here all those with whom employees interact in their internal everyday work are considered. Therefore, the phrase “In daily collaboration” can be found at the beginnings of the items. Furthermore, the explanation “In the following questions, please refer to your leaders, co-workers, etc., and thus to all those involved in your daily work” is included. Another difference is that significantly fewer items are used than in the HoL in order to achieve greater practical applicability.

Three items from the areas of value and awareness selected from the HoL and four items from the area of behavior were used since the original questionnaire also elicits behavior with a higher proportion of statements. The items were rephrased to ask for health-promoting collaboration and not health-oriented leadership. For example, the item “My leader notices immediately if something is wrong with my health” was changed to “In daily collaboration it is noticed immediately if something is wrong with my health.” For the area of behavior, the statement “In daily collaboration I receive support when needed” was added as an additional item independent of the HoL. The response options from 1 = “does not apply” to 5 = “applies” were also taken from the HoL.

### Statistical Analyses

IBM SPSS Statistics software was used in the statistical analysis of the data. Most of the scales achieved adequate values for internal consistency (Table 1).

**TABLE 1:** Descriptive statistics and reliability of the scales.

Scale	Number of items	Scaling	*M*	*SD*	*Min*	*Max*	Cronbach’s alpha
Health-promoting collaboration	11	1–5	3.04	0.74	1.09	5.00	0.86
Value	3	1–5	3.26	0.96	1.00	5.00	0.69
Awareness	3	1–5	2.98	0.93	1.00	5.00	0.72
Behavior	5	1–5	2.92	0.81	1.00	5.00	0.79
Presenteeism	5	1–3	1.54	0.49	1.00	3.00	0.75
Well-being	5	1–6	3.74	1.06	1.00	6.00	0.89
Work ability	4	1–5	3.90	0.86	1.00	5.00	0.82

*N = 303–308. M, Mean; SD, Standard deviation; Min, Minimum; Max, Maximum.*

The correlations assumed for Hypotheses 1–3 were tested using Pearson’s product-moment correlation coefficient. The influence of presenteeism on the presumed relationships between health-promoting collaboration and well-being or work ability was tested by mediator analyses using Hayes’ PROCESS macro. Here, health-promoting collaboration was the predictor, well-being or work ability the criterion, and presenteeism the mediator. The confidence interval was set at 95% based on 10,000 bootstrapping samples. The variables were centered on the mean value.

## Results

### Descriptive Analyses

Table 1 summarizes the descriptive statistics and the reliability of the scales used. With regard to the extent of health-promoting collaboration, a divided picture is shown descriptively. The mean value of the 5-point scale is 3.04 (*SD* = 0.74), whereby a high value stands for a highly pronounced health-promoting collaboration. The individual analysis of the three components value (importance of health in daily collaboration), awareness (mutual conscious perception in daily collaboration), and behavior (mutual active care for health in daily collaboration) reveals slight differences. Value is rated highest with a mean of 3.26 (*SD* = 0.96), followed by awareness (*M* = 2.98; *SD* = 0.93) and behavior (*M* = 2.92; *SD* = 0.81).

The mean value of presenteeism is 1.54 (*SD* = 0.49), indicating that the majority of respondents exhibited presenteeism “not once” to “once” in the last year. With regard to this mean, however, it should be noted that the extreme items, such as “In the last 12 months, how often did you take vacation days for recovery?” are also included here.

In terms of well-being, the mean score is on the average level (*M* = 3.74; *SD* = 1.06) and work ability is rated above average (*M* = 3.90; *SD* = 0.86).

### Correlation Analyses

Table 2 represents the correlation matrix of the variables investigated. The relationships emerging provide answers to Hypotheses 1–3. Small to medium effects are shown. Health-promoting collaboration is statistically significantly negatively related to presenteeism (*r* = −0.18, *p* ≤ 0.01). Consequently, high health-promoting collaboration is associated with lower presenteeism. The first hypothesis can be confirmed.

**TABLE 2:** Intercorrelations of the variables.

		1	2	3	4
1	Health-promoting collaboration	1			
2	Presenteeism	−0.18**	1		
3	Well-being	0.32***	−0.31***	1	
4	Work ability	0.26***	−0.26***	0.56***	1

N = 307–308, ***p < 0.001, **p < 0.01.

Furthermore, health-promoting collaboration shows statistically significant positive relationships with well-being (*r* = 0.32, *p* ≤ 0.001) and work ability (*r* = 0.26, *p* ≤ 0.001). Hypotheses 2 and 3 can thus also be supported. If the collaboration is perceived as health-promoting, then both well-being and the work ability are increased.

Beyond the hypotheses, Table 2 shows that well-being and work ability are statistically significantly negatively related to presenteeism and statistically significantly positively correlated with each other.

### Mediator Analyses

Hypotheses 2a and 3a are intended to test whether the relationships between health-promoting collaboration and both well-being and work ability can be explained, at least in part, by presenteeism.

The direct effect describes the effect of the predictor (health-promoting collaboration) on the criterion (well-being/work ability) with simultaneous consideration of the mediator (presenteeism). The indirect effect describes the effect of the predictor on the criterion via the mediator. Direct effect and indirect effect together result in the total effect (Hayes, 2013).

#### Well-Being

**FIGURE 1:**
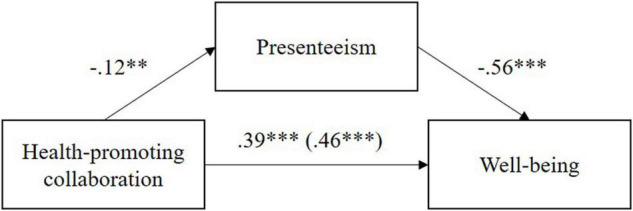
Presentation of the *B*-values of the mediator analysis with the criterion well-being. The *B*-value of the total effect is given in parentheses. ***p < 0.001, **p < 0.01.

The results of the mediator analysis for the criterion well-being are presented in the following. Figure 1 shows that an increase in health-promoting collaboration predicts a decrease in presenteeism, which in turn predicts an improvement in well-being.

Table 3 shows the regression coefficients of the total, direct, and indirect effect. The indirect effect is of particular relevance for testing mediation. It can be used to assess whether the relationship between health-promoting collaboration and well-being is partially mediated by presenteeism. This indirect effect (*B* = 0.07, *SE* = 0.03) is significant (95% BC CI [0.02, 0.13]) because zero is not included in the confidence interval. Consequently, presenteeism is partially (albeit with a small effect) an underlying mechanism explaining the relationship between health-promoting collaboration and well-being. The effect postulated in Hypothesis 2a could be confirmed.

**TABLE 3:** Total, direct, and indirect effect of health-promoting collaboration and presenteeism on well-being.

	*B*	*SE B*	*t*	Bootstrapping		*R* ^2^
				95% BC CI		
				Lower	Upper	
Total effect	0.46	0.08	5.89***	0.31	0.62	0.10***
Direct effect	0.39	0.08	5.12***	0.24	0.54	
Indirect effect	0.07	0.03		0.02	0.13	
		*F*_(1, 305)_ = 34.63, *p* < 0.001				

N = 307, ***p < 0.001. B, regression coefficient; SE B, standard error; BC CI, bias-corrected confidence interval; R^2^, explained variance.

#### Work Ability

**FIGURE 2:**
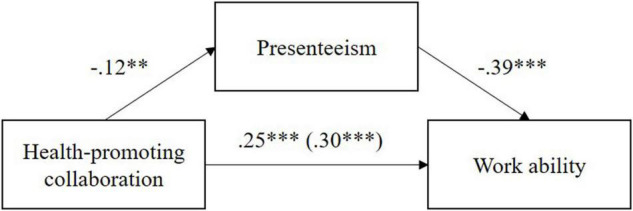
Presentation of the *B*-values of the mediator analysis with the criterion work ability. The *B*-value of the total effect is given in parentheses. ***p < 0.001, **p < 0.01.

The results of the mediator analysis for the criterion work ability are presented in the following. Figure 2 shows that an increase in health-promoting collaboration predicts a decrease in presenteeism, which in turn predicts an improvement in work ability.

Table 4 shows the regression coefficients of the total, direct and indirect effect. The indirect effect (*B* = 0.05, *SE* = 0.02) is significant (95% BC CI [0.01, 0.09]) because zero is not included in the confidence interval. Consequently, presenteeism is partially (albeit with a small effect) an underlying mechanism explaining the relationship between health-promoting collaboration and work ability. Hypothesis 3a is supported.

**TABLE 4:** Total, direct, and indirect effect of health-promoting collaboration and presenteeism on work ability.

	*B*	*SE B*	*t*	Bootstrapping		*R* ^2^
				95% BC CI		
				Lower	Upper	
Total effect	0.30	0.06	4.68***	0.17	0.43	0.07***
Direct effect	0.25	0.06	3.98***	0.13	0.38	
Indirect effect	0.05	0.02		0.01	0.09	
		*F*_(1, 305)_ = 21.86, *p* < 0.001				

N = 307, ***p < 0.001. B, regression coefficient; SE B, standard error; BC CI, bias-corrected confidence interval; R^2^, explained variance.

## Discussion

So far, little research has been presented on concrete measures to reduce presenteeism. Therefore, the conducted study can offer an important contribution to presenteeism research. The starting point of the study was the finding of previous studies that how an organization deals with health influences whether presenteeism occurs or not (Wieland and Hammes, 2010; Chen et al., 2015; Hägerbäumer, 2017; Ruhle and Süß, 2020; Komp et al., 2021). We assumed that not only a healthy leadership is a requisite for a culture of health but also daily collaboration among co-workers. We propose two main reasons for this: Firstly, the increase in teleworking and virtual teams entails more virtual exchanges (Ohlbrecht and Seltrecht, 2018) and secondly, shared leadership (Zhu et al., 2018). Therefore, in modern forms of collaboration (digital or shared leadership), the leader can no longer be solely responsible for the health of employees. In light of these findings, the concept of health-promoting collaboration was developed, which, in addition to the leader, includes co-workers. The basis for formulating the items was the HoL (Pundt and Felfe, 2017).

In the first step, a negative relationship between health-promoting collaboration and presenteeism was assumed. Since health-promoting collaboration is an extension of healthy leadership, we considered the influence of leadership when deriving the hypotheses. Leaders have an influence on the behavior of employees in case of illness mainly due to their function as role models (Dietz et al., 2020; Komp et al., 2021). This function can be transferred to co-workers, who can also be seen as role models. Additionally, employees can address their peers when these exhibit presenteeism, possibly resulting in a reduction in this behavior. The assumed relationship between health-promoting collaboration and presenteeism was significant, confirming the conjectures in line with the findings on healthy leadership (Dietz et al., 2020; Komp et al., 2021).

In addition to this relationship, we also investigated whether health-promoting collaboration affects the constructs of well-being and work ability, which have been widely studied in the work context. Studies on healthy leadership show that leadership behavior can contribute to improved well-being (Franke et al., 2014; Rigotti et al., 2014; Santa Maria et al., 2019) and work ability (Vincent, 2011). Building on these findings, these relationships were likewise hypothesized to apply to health-promoting collaboration. One worker can improve co-workers’ well-being by taking over some of their tasks during stressful work periods (Osterspey, 2012). An improvement in work ability is possible when co-workers support each other in work-related problems or share their knowledge as part of teamwork (Osterspey, 2012). Correlations between health-promoting collaboration and both well-being and work ability were shown.

In the last step, we examined whether presenteeism mediates the relationships between health-promoting collaboration and well-being and work ability. This mediator effect was suspected since both presenteeism and health-promoting collaboration are related to well-being and work ability, and health-promoting collaboration is assumed to influence presenteeism. The mediator analyses yielded significant results. Although the indirect effects are small, the result shows that presenteeism does indeed exert a mediating influence. This influence means that presenteeism occurring due to poor health-promoting collaboration contributes to a reduction in well-being and work ability. Thus, the results of Baeriswyl et al. (2017) could be transferred to the context of health-promoting collaboration. Baeriswyl et al. (2017) showed that the effect of co-worker support on emotional exhaustion is mediated by presenteeism. Instead of co-worker support, the present study examined health-promoting collaboration and, instead of emotional exhaustion, the more general constructs of well-being and work ability. The identification of presenteeism as a mediator affords a better understanding of the effects of health-promoting collaboration on well-being and on work ability.

### Limitations and Future Research

Despite the strengths of the study, which are primarily the investigation of the innovative concept of health-promoting collaboration in combatting presenteeism, there are limitations. To develop the health-promoting collaboration scale, part of the HoL (Pundt and Felfe, 2017) was shortened and only slightly adapted by removing the focus on leaders. Nevertheless, the whole scale should be validated and possibly revised in the future. The entire concept of health-promoting collaboration should be explored further, especially with regard to modern work and leadership structures.

Some researchers, such as Maxwell and Cole (2007), judge the use of mediation to be critical in cross-sectional data. However, we follow Hayes (2013), who reasons that mediation can be conducted as long as the data are interpreted with caution. Fairchild and McDaniel (2017) likewise state that the calculation is possible as long as the assumed temporal order of predictor, mediator, and criterion can be well justified, which is the case in this study. Preliminary results suggest that health-promoting collaboration has an influence on presenteeism (Hägerbäumer, 2017; Komp et al., 2021). The influence of presenteeism on well-being and work ability has been shown in longitudinal studies (Dellve et al., 2011; Gustafsson and Marklund, 2011). Despite the theoretical derivation of the direction of causality in future collecting longitudinal data will be advisable to confirm the results obtained.

Another limitation is that only the university context was investigated. However, both academic staff and professors as well as administrative staff with heterogeneous leadership structures were included in the survey. This mix renders feasible generalization to different forms of leadership, also those outside academia. It is only relevant that the corresponding work contexts have similar working conditions, especially with regard to leadership constellations. The trends of digitalization and shared leadership, which are the base for developing the concept of health-promoting collaboration and which entail new forms of leadership, also occur outside the university setting.

### Practical Implications

It became apparent that, in addition to the leaders, co-workers are of great importance in establishing a culture of health. Employees must be made aware of their role in the health of their co-workers. Implications can be given for all three facets of health-promoting collaboration: On the dimension of value, workshops in which both leaders and employees are made aware of the relevance of health are an appropriate option (Felfe, 2015). Concerning awareness, various awareness-raising practices are possible in which techniques are acquired that help employees to become more aware of their co-workers’ state of health (Felfe, 2015). The goal should be to recognize co-workers’ complaints or stress in order to provide support and alleviate burdens at the behavioral level in the next step. At the behavioral level, it also makes sense to promote positive interaction among co-workers or to motivate them to participate in workplace health promotion programs. All these interventions strengthen health-promoting collaboration, which can contribute to reducing presenteeism. Leaders noticing high presenteeism in their units might approach the issue of health-promoting collaboration. Better health-promoting collaboration additionally leads to better well-being and work ability. If all employees join forces in establishing a culture of health, this will relieve the burden on leaders.

The consideration of presenteeism is particularly interesting against the background of the current trend toward increased teleworking. In home offices, the barriers to working when sick are lower, and hence conducive to presenteeism (Steidelmüller et al., 2020). Organizations need to be aware of the risks of presenteeism in the home office and should design telework so as to avoid behavior detrimental to health (Steidelmüller et al., 2020). It can be assumed that health-promoting collaboration plays a major role here.

## Conclusion

The study identified the concept of health-promoting collaboration as a starting point for reducing presenteeism, thus providing important guidance for organizations on dealing with presenteeism. The newly developed concept includes not only leaders but also co-workers, which is particularly relevant in modern forms of collaboration (digital and shared leadership). In addition to the effects on presenteeism, there are positive effects on well-being and work ability. Furthermore, presenteeism was found to mediate the relationship between health-promoting collaboration and both well-being and work ability.

The results thus provide further insights for a more comprehensive understanding of presenteeism and, above all, ways to reduce its negative effects. In practice, various starting points could be identified within the context of the three dimensions of health-promoting collaboration. One example is to provide workshops to sensitize participants to the subject of health. The scale for measuring health-promoting collaboration should be validated and longitudinal studies should be conducted to test the mediation model in particular. The relationship between presenteeism and health-promoting collaboration should be further investigated, especially against the background of modern, digital forms of work and in other contexts, also outside academia.

## Data Availability Statement

The datasets presented in this article are not readily available because the data were collected in a public institution (university). The dataset can be made available in anonymized form under the condition that the university agrees. Requests to access the datasets should be directed to RK, rebecca.komp@h-brs.de.

## Author Contributions

RK and PI-D organized the database. RK performed the statistical analysis and wrote the first draft of the manuscript. All authors contributed to manuscript revision, read, and approved the submitted version, and contributed to conception and design of the study.

## Conflict of Interest

The authors declare that the research was conducted in the absence of any commercial or financial relationships that could be construed as a potential conflict of interest.

## Publisher’s Note

All claims expressed in this article are solely those of the authors and do not necessarily represent those of their affiliated organizations, or those of the publisher, the editors and the reviewers. Any product that may be evaluated in this article, or claim that may be made by its manufacturer, is not guaranteed or endorsed by the publisher.

## Footnotes

1 In the context of healthy leadership, various constructs can be found, such as health-promoting or health-oriented leadership (Rudolph et al., 2020). Due to the similarity of the constructs, no distinction is made here; we will speak of healthy leadership in general.
